# Acute Metastatic Spinal Cord Compression: Urgent Surgery versus Radiotherapy and Treatment Result Prediction versus Actual Results

**DOI:** 10.3390/curroncol29100583

**Published:** 2022-10-05

**Authors:** Oded Hershkovich, Mojahed Sakhnini, Sharif Gara, Israel Caspi, Raphael Lotan

**Affiliations:** 1 Department of Orthopedic Surgery, Wolfson Medical Center, Ha-Lokhamim St. 62, Holon 5822012, Israel; 2 Department of Orthopedic Surgery, Sheba Medical Center, Tel Aviv University, Tel Aviv 6997801, Israel

**Keywords:** cord, compression, radiation, metastasis

## Abstract

**Background Context:** The role of radiotherapy versus surgery in treating acute metastatic spinal cord compression (AMSCC) has changed over the years. **Purpose:** Our study evaluates neurological and functional outcomes following urgent surgery and radiotherapy (USFR) versus urgent radiotherapy alone in treating AMSCC. **Study Design/Setting:** A retrospective cohort of 54 patients with AMSCC with variable neurological deficits. Overall, 32 patients were treated with USFR, and 22 received urgent radiotherapy alone. **Outcome Measures**: Neurological status regarding the Asia and Frankel scores, continence and ambulation, and Kranofsky’s functional score and patient life span comprised the outcome measures. **Methods:** This was a retrospective EMR study. **Results:** USFR and radiotherapy cohorts were similar in age, gender, tumor origin, and the number of spinal metastases. The most common cause of AMSCC was carcinoma of the breast (24.1%), followed by carcinoma of the lung (16.7%) and multiple myeloma (13%). Neurological status at AMSCC presentation was similar between cohorts regarding Asia and Frankel scores, continence and ambulation, and Kranofsky’s functional score. Following USFR, 59.3% of the patients had a motor strength improvement, 31.3% regained sphincter function, and 34.4% regained ambulation, while 90% of the patients treated by radiotherapy did not show any improvement. One patient under radiotherapy lost sphincter function. The treatment received did not affect the patient’s survival. A subanalysis of patients with a short life expectancy, by Tomita and Tokuhashi scores, showed missed prediction in 29.4% of cases. **Conclusion:** The study supports the beneficial effect of UFSR compared to urgent radiotherapy alone in treating AMSCC in all subgroups. Early surgery improved function, motor strength, sphincter control, and ambulation without affecting life span. Prognostic scores failed to predict life span in almost one-third of the patients, requiring further investigation.

## 1. Introduction

Acute metastatic cord compression (AMSCC) is an epidural metastatic lesion displacing and compressing the spinal cord within the spinal canal [1]. It is a significant source of morbidity, causing paralysis and incontinence [2]. AMSCC is estimated to occur in up to 10% of patients with cancer, most commonly originating in the breast, prostate, and lung cancers. Up to 40% of patients with AMSCC have concomitant bone metastases outside the spine [3,4].

AMSCC presents as a neurological deterioration, insidious or abrupt, necessitating urgent diagnosis and treatment. In some patients, AMSCC can even be the presenting symptom of an undiagnosed malignancy [5,6]. The neurological deterioration can affect the patient’s ability to ambulate and function independently with variable loss of sphincter control. Although the degree of clinical progression is inconstant, patients with motor dysfunction unavoidably progress to complete paralysis without intervention [7]. Nearly half of AMSCC patients cannot walk by the time of diagnosis [8]. Neurological status at the diagnosis, predominantly motor function deficits, is directly associated with prognosis from metastatic cord compression [9], emphasizing the importance of early diagnosis before the patient develops a neurological deficit.

Studies have shown that urgent treatment before paralysis is clinically efficacious and cost effective [9]. Spinal metastases can cause mechanical instability, leading to neurological injury or direct metastatic cord compression due to epidural involvement. The epidural involvement also compresses the venous plexus, causing edema that aggravates the compressive effect on the suffering spinal cord. That explains the narrow time frame for treatment to prevent irreversible damage to the spinal cord. Surgery directly decompresses the spinal cord and stabilizes the spine. Radiotherapy alone can allow for local tumor control, reduce metastasis size, and, combined with steroids, can decrease the edema surrounding the spinal cord [10,11]. Studies claim that radiotherapy’s effect is slow and cannot save the compromised cord in the relevant time frame. Urgent direct decompressive surgery with stabilization was advantageous in achieving functional improvement [12]. Although the recent literature recommends urgent surgery followed by radiotherapy as the preferred approach to AMSCC [13], controversy still exists regarding timing and optimal treatment. The AMSCC patient cohort is very heterogenic in ambulatory status, baseline function, malignancy histology, age, the timing of diagnosis, neurological status at diagnosis, metastatic system burden, tumor genetics, and other factors investigated in multiple studies creating ambiguity regarding patient-specific optimal treatment [14,15,16].

AMSCC studies initially evaluated non-instrumented decompressive laminectomies with reports of equal effectiveness to radiotherapy, excluding the operative complications [10,11,12]. Publications evaluating tumor debulking with spinal stabilization showed superior outcomes compared to radiotherapy alone. Patchell’s [13] study was the first randomized control trial to examine AMSCC and is highly acclaimed and cited, comparing surgical decompression and radiation therapy with superior outcomes following surgery. Patchell’s findings were supported by Kim et al. and Lida et al. [14,15]. However, it is criticized [12,14,16] for excluding patients with radiosensitive tumors, the small sample size, and methodological problems, including selection bias. Rades’s [16] study, a matched-pair analysis of 324 patients, failed to demonstrate an advantage of surgery over radiation therapy and called for a new randomized trial.

This study compares the results of urgent surgery for AMSCC to radiotherapy alone regarding neurological status, continence, ambulation, and mortality.

## 2. Methods

A retrospective study included patients who had urgent surgery or urgent radiotherapy for AMSCC between 2009 and 2017. Patients’ EMR was evaluated for age, gender, ASIA and Frankel scores on admission and at follow-up, the timing of surgery (within 48 h of admission), ambulatory status, bowel and bladder continence, rectal tone and voluntary squeeze on physical exam, medical history, and the tumor’s histological type, as well as the number and location of spinal, osseous, and soft tissue metastases as they appeared on MRI, CT scans, bone scans, and PET-CTs. Patient’s Karnofsky, Tomita, and Tokuhashi scores were calculated. Patient death was recorded up to the end of 2018.

AMSCC diagnosis was confirmed on a whole-spine MRI. Upon diagnosis, treatment was decided by a multidisciplinary team, a neurosurgeon or orthopedic spine consultant who were on call and a radiation oncologist, and surgery followed by radiotherapy or immediate radiotherapy. Radiation therapy consisted of a pre-set protocol, supervised by a radiation oncologist, with patients receiving 30 Gy in ten 3D fractions, with the first treatment performed within 72 h of AMSCC presentation.

Exclusion criteria included patients treated in other medical centers and primary bone tumors of the spine. No patients were lost to follow-up.

Our institutional review board approved the study.

Study data statistical analyses were carried out using SPSS 20.0. Numerical variable differences were calculated using the Student’s *t*-test. Categorical parameters were assessed using the Chi-square test or Fisher exact tests. Kaplan–Meier survival curves were used to compare survival between the two cohorts. The survival curves were compared using the log-rank test. Cox regression was used to evaluate the effect of different covariates on survival.

## 3. Results

Fifty-four patients were diagnosed with acute metastatic cord compression (AMSCC), ranging from complete paraplegia to variable paraparesis. Thirty-two patients underwent urgent surgery within 48 h of hospitalization, and twenty-two patients were treated by radiotherapy alone. Cohorts were similar in age, gender, tumor origin, and the number of spinal metastases (Table 1). The radiotherapy group had a significantly higher osseous disease load (*p* = 0.004) and a longer period from tumor diagnosis to AMSCC (*p* = 0.02). The cohorts did not differ regarding soft tissue disease load (*p* = 0.378) (Table 1).

The most common cause of AMSCC was carcinoma of the breast (24.1%), followed by carcinoma of the lung (16.7%) and multiple myeloma (13%) (Table 1). Osseous metastases most commonly involved the pelvis (26%), chest wall (22%), and upper limb (16.7%) (Table 2). Femoral metastases were noted in 15% of the cases, warranting a specific workup to identify imminent fractures. Lungs and lymph nodes accounted for more than half of the extra-skeletal metastatic disease (52.8%) (Table 2).

Neurological status at AMSCC presentation was similar between cohorts regarding Asia and Frankel scores, continence, and ambulation (0.1 < *p* < 0.7) (Table 1). Kranofsky’s functional score was similar between the cohorts (*p* = 0.16), but the prognosis predictive average Tomita score was significantly lower for the surgical cohort (4.9 ± 2.1 versus 6 ± 1.9, *p* = 0.04). In comparison, the average Tokuhashi score showed a trend toward higher scores in the surgical group (7.8 ± 2.3 versus 6.5 ± 2.3, *p* = 0.053).

**Figure 1:**
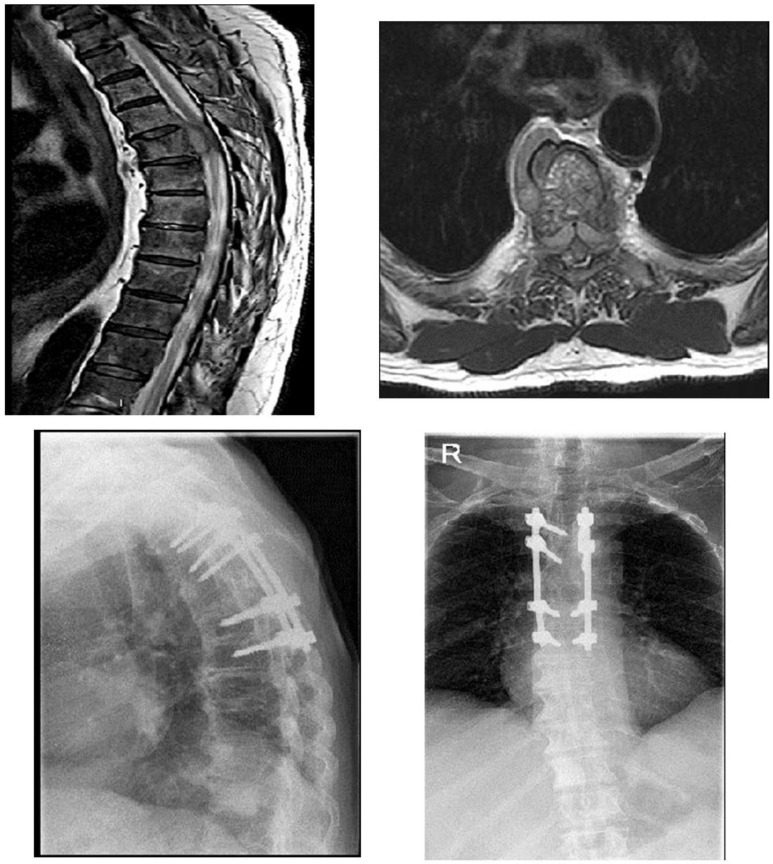
A 78-year-old patient presented to the ER with variable weakness to lower limbs and loss of ability to ambulate. MRI studies revealed a compressing metastasis in D5 as shown on the sagittal and axial T2 images. The patient underwent urgent surgical decompression with partial corpectomy of D5 and stabilization with pedicular screws for two levels above and two levels below the involved lesion. The biopsy revealed a prostate source. Full neurological recovery of motor function was achieved following the surgery.

**Figure 2:**
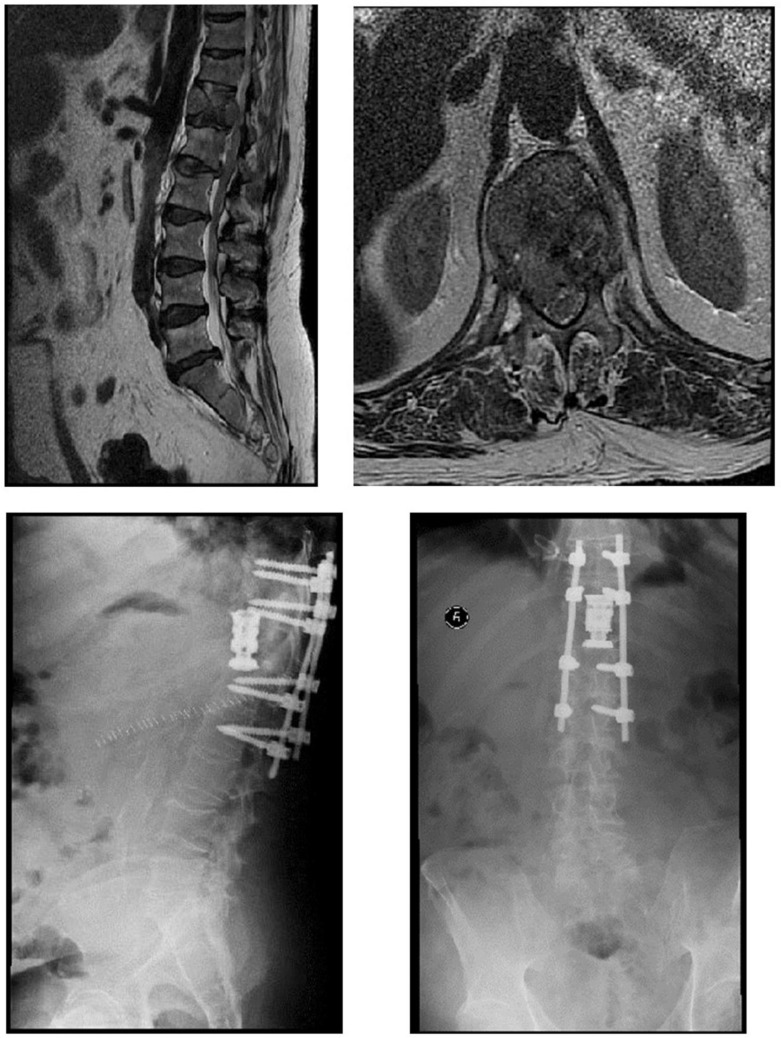
A 57-year-old patient presented to the ER with deterioration in neurological status in his lower limbs and loss of ability to ambulate. MRI sagittal and axial T2 studies revealed a metastatic lesion in D12 with mechanical pressure on his cord. Urgent surgery was performed with a double approach: a posterior approach for decompression of the cord, and stabilization with pedicular screws for two levels above and two levels below D12. Next, a thoracotomy was performed for a complete corpectomy of D12 and the utilization of a cage. After the surgery, the patient regained the ability to ambulate with complete recovery from neurological deterioration. A pathological specimen revealed a liver source for metastasis.

Thirty-two patients underwent urgent surgery for AMSCC, including posterior decompression and stabilization (Figure 1 and Figure 2), and twenty-two patients underwent radiation therapy. Radiation therapy consisted of a pre-set protocol, supervised by a radiation oncologist, with patients receiving 30 Gy in ten 3D fractions.

**Figure 3:**
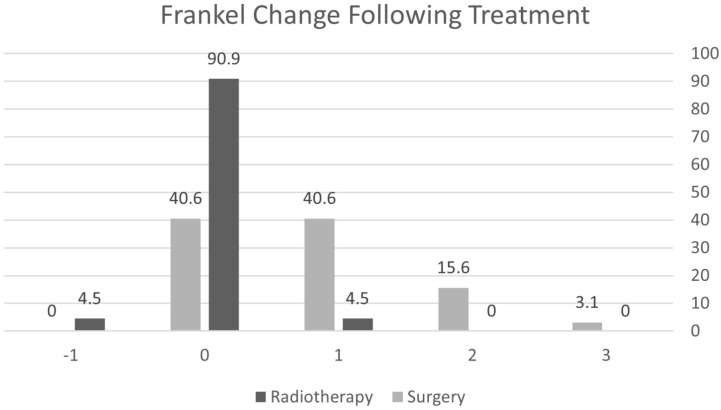
Change in Frankel score following treatment.

Following surgery, 59.3% of the patients had a motor strength improvement on Frankel grading, most (40.6%) by one grade (Figure 3). In total, 90% of the patients treated by radiotherapy alone for AMSCC did not show any improvement in motor strength, and 4.8% even deteriorated. The Karnofsky functional score improved by 11 points following surgery (*p* = 0.016), while it did not change significantly under radiotherapy (*p* = 0.466). The Karnofsky change following treatment was significantly better following surgery (*p* < 0.0001) while not improving under radiotherapy. Eleven surgery patients (34.4%) regained ambulatory status; they presented to the emergency department non-ambulating and regained ambulation following surgery. None of the patients regained ambulation following radiotherapy (Table 3).

Sphincter function in both treatment groups, pre- and post-intervention, was evaluated, and significantly more patients in the surgery group regained sphincter function than in radiotherapy (*p* < 0.0001). In total, 68.8% of the surgery cohort had no change in sphincter function, either remaining continent or incontinent, compared to 81.8% in the radiotherapy cohort. Additionally, 31.3% of patients regained sphincter function following surgery compared to 13.6% under radiotherapy (*p* < 0.0001) (Table 4). One patient under radiotherapy lost sphincter function.

In months, the time of AMSCC to death was not statistically different between the two groups (0.348). AMSCC was the presenting symptom of malignancy for 11 patients in the surgery group and 3 in the radiation group (34.4% and 13.6%, respectively). Consequently, the average time from cancer diagnosis to AMSCC was significantly different between surgery and radiotherapy cohorts (2.5 ± 5 and 6.9 ± 5.9 years, respectively, *p* = 0.005).

**Figure 4:**
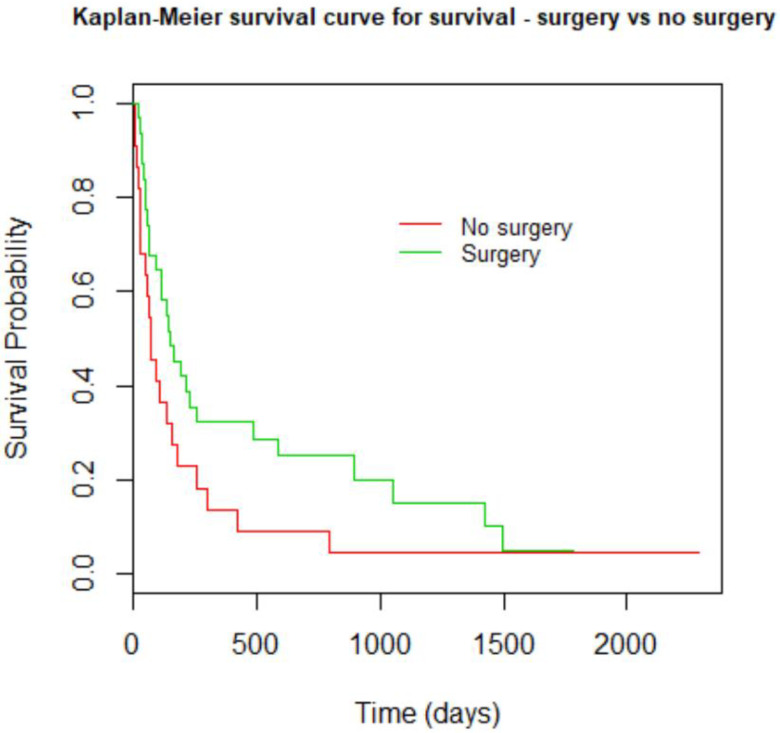
Kaplan–Meyer curve compares the survival of both groups of treatment: surgical intervention vs. radiation therapy alone. *p* = 0.0765.

**Figure 5:**
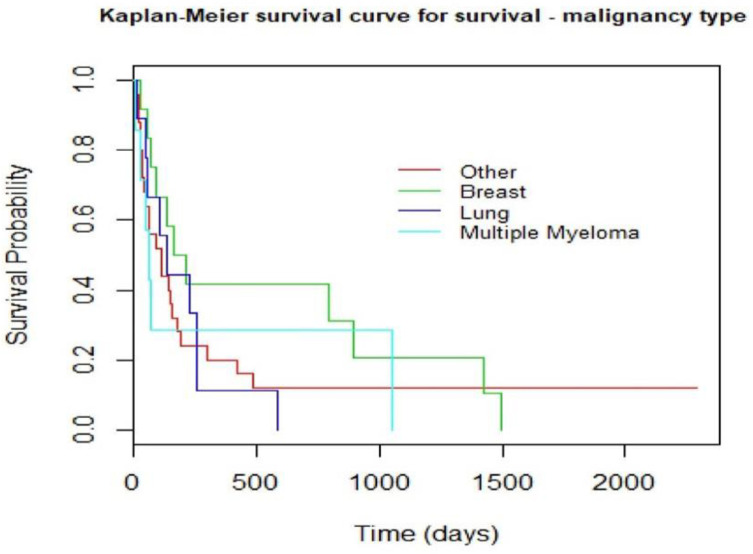
Kaplan–Meyer curve that compares survival among the most frequent malignancies in the study. *p* = 0.64.

THE Kaplan–Meier survival curve analysis comparing surgery to radiotherapy showed a trend toward better survival in the surgery group but did not reach statistical significance (Figure 4). In this study, the Kaplan–Meier curve comparing the different malignancies causing AMSCC showed that breast carcinoma patients had better survival than lung cancer, although this was not statistically significant (Figure 5).

A further subanalysis was performed on patients with a short life expectancy, i.e., a Tomita score >7 or a Tokuhashi score of <9. This group included 19 patients from the surgery cohort and 15 from the radiotherapy cohort. These subgroups were similar regarding age (57.6 ± 17.9 vs 60.5 ± 15.6, respectively, *p* = 0.623), gender (*p* = 0.42), and neurological status (0.228 < *p* < 0.70), as well as the Tomita, Tokuhashi, and Karnofsky scores (0.084 < *p* < 0.19). The only statistically significant difference was the time difference between malignancy diagnosis and AMSCC, 1.6 ± 2.5 years for the surgery subgroup and 6.3 ± 4.8 years for the radiotherapy subgroup (*p* = 0.0008). The whole short life expectancy subgroup had Tokuhashi scores less than 9, meaning a life expectancy shorter than six months. Still, seven patients of the surgery subgroup survived longer (18.7 ± 13.4 months) than expected compared to only three patients in the radiotherapy subgroup (10 ± 2.6 months) (*p* = 0.123). Five patients of the short life expectancy subgroup had a Tomita score of 9 and 10, meaning a life expectancy shorter than three months, but three patients survived for four, eight, and nine months, respectively, while the other two died within a month.

## 4. Discussion

Accurate diagnosis and treatment of patients with AMSCC is a pivotal event and can change the quality of life and survival. Early diagnosis and treatment of AMSCC are essential to prevent permanent neurological damage, so early recognition coupled with rapid referral pathways and treatment is required. Recently, the treatment paradigm shifted from radiation-only therapy to a more aggressive approach combining surgery with other modalities [14]. The change was influenced by advances in surgical techniques, leading to better outcomes than radiotherapy alone [5].

In our study, surgery within 48 h of AMSCC and urgent radiotherapy cohorts had similar characteristics, including the type of malignancy and neurological status, as evaluated by ASIA and Frankel scores, continence, ambulatory status, and the Karnofsy scale (Table 1). Prognostic scores were also comparable; the Tokuhashi score tended to be worse in the radiation alone group (a difference of 1.3 points on a 15-point scale, *p* = 0.053), as did the Tomita scores (a difference of 1.1 points on a 10-point scale, *p* = 0.04). Those mild differences between the groups lack clinical significance. There were more patients with AMSCC as the presenting symptom of malignancy in the surgical group (34.4%) than in the radiation-alone group (13.6%), explaining the shorter median time from cancer diagnosis to cord compression in the surgery group.

The study cohorts were not randomized, and a selection bias exists regarding time with a diagnosed malignancy (*p* = 0.005) and the number of extra-spinal osseous metastases (*p* = 0.004), favoring radiotherapy. However, the cohorts did not significantly differ between functional and prognostic scores, probably reducing the impact of additional osseous metastatic involvement on patient survival.

In our study, surgery remains a superior treatment option compared to radiotherapy to maintain or improve motor strength, ambulation, and sphincter control (Table 3 and Table 4, Figure 3), adding to the quality of life [13,14,15,17]. Radiotherapy might have prevented further neurological deterioration in most AMSCC patients, but none improved under treatment [15,18,19,20,21]. Surgery resulted in a significantly improved Karnofsky functional score compared to radiotherapy, which did not improve function (*p* < 0.0001).

Rades’s study [16] divided AMSCC patients into those with motor deficits for up to a week and more than a week; this division shifted the results toward disadvantageous surgical outcomes, as surgery was performed following irreversible neurological damage. Our study confirms that surgery within 48 h of AMSCC provides better neurological outcomes than radiotherapy alone. This study did not examine the results of delayed surgery, as it is against our department’s policy.

Malignancy’s origin and treatment modality did not affect our series’ survival (Figure 4 and Figure 5). According to prior studies [17], breast carcinoma is the most prevalent malignancy causing AMSCC, followed by lung and multiple myeloma, similar to our cohort. The development of cord compression is universally associated with a shorter life span; as evident in our series, the median time to death was 6.4 months in the radiation group and 9.9 months in the surgery group (*p* = 0.347). Although surgery did not affect survival significantly in this study, the impact on function, motor strength, sphincter control, and ambulation is substantial.

Since Patchell’s study [13], there have been significant advances in oncological treatment options: biological therapy, immunotherapy, and radiotherapy [22]. These advances probably change the ability of the current prognostic scales, Tomita and Tokuhashi, to predict AMSCC patients’ life spans. In our study, 29.4% of the short life expectancy subgroup survived longer than anticipated, suggesting a high error margin. These results warrant a more aggressive approach to surgery, raising the threshold for radiotherapy alone. Further studies are required to evaluate the role of the current prognostic scales in light of the new era of oncological treatments available.

This study revalidates the evidence that urgent surgery is superior to radiation treatment. Surgery in our cohort reversed neurological deterioration, paraplegia, and bowel and bladder control loss among patients with AMSCC, even regaining ambulation compared to radiotherapy alone.

## 5. Conclusions

The study supports the beneficial effect of urgent surgical intervention compared to radiotherapy alone in treating AMSCC in all subgroups. Early surgery improved function, motor strength, sphincter control, and ambulation without affecting life span. Prognostic scores failed to predict life span in almost one-third of the patients, requiring further investigation.

## Figures and Tables

**Table 1:** Baseline characteristics of patients.

	Radiation Group (22)	Surgery Group (32)	*p* Value
Mean age (years)	60.8 ± 15.9	59.6 ± 14.2	0.781
Male	10 (32.3%)	13 (41.9%)	0.835
Primary tumors			0.614
Breast	27.3%	21.9%	
Lung	18.2%	15.6%	
Multiple Myeloma	18.2%	9.4%	
Prostate	4.5%	9.4%	
Hepatic	4.5%	6.3%	
Melanoma	9.1%	3.1%	
Bladder	4.5%	3.1%	
Other	13.7%	31.2%	
Time from tumor diagnosis to AMSCC (y)	6.9 ± 5.9	2.5 ± 5	0.005
Number of spinal metastases	2.9 ± 1.4	2.3 ± 1.3	0.1546
Osseous metastases other than spine	1.4 ± 1.4	0.5 ± 0.7	0.004
Soft tissue metastases	1.3 ± 1.1	0.9 ± 1.5	0.378
Average initial Frankel score	C	C	0.4
Average initial ASIA score	83.7 ± 17.1	75.6 ± 19.5	0.12
Ambulatory at AMSCC presentation	9 (40.9%)	10 (31.3%)	0.721
Continent at AMSCC presentation	10 (45.5%)	17 (53.1%)	0.693
Initial Karnofsky score	51.8 ± 24.8	46.3 ± 14.8	0.306
Average Karnofsky score post-treatment	46.8 ± 20.1	57.5 ± 21.1	0.068
Tokuhashi score	6.5 ± 2.3	7.8 ± 2.3	0.053
Tomita score	6 ± 1.9	4.9 ± 2.1	0.04
Average time to death (months)	6.4 ± 12.8	9.9 ± 14	0.347
Death within 30 days	7 (31.8%)	2 (6.3%)	0.04
Death within six months	17 (77.3%)	16 (50.0%)	0.11

**Table 2:** Non-spinal metastatic disease distribution at AMSCC onset.

Non-Spinal Metastatic Disease Distribution at ASMCC Onset			
Osseous		Soft Tissue	
**Pelvis**	25.9%	Lung	25.9%
**Chest wall**	22.2%	Lymph nodes	25.9%
**Upper limb**	16.7%	Brain	16.7%
**Femur**	14.8%	Mediastinum	7.4%
**Multiple**	5.6%	Retroperitoneum	7.4%
**Other locations**	20.4%	Other locations	16.7%

**Table 3:** Change in ambulation following treatment.

Radiotherapy (%)	Surgery (%)	
**0.0**	34.4	Gained ambulation
**90.9**	65.6	No change
**9.1**	0.0	Lost Ambulation

**Table 4:** Sphincter function changes following the intervention.

Radiotherapy(%)	Surgery (%)	
**81.8**	68.8	No change
**13.6**	31.3	Gained Sphincter
**4.5**	0	Lost sphincter

## Data Availability

The complete data are available under a confidentiality restriction.
